# The Science (or Nonscience) of Research Into Sudden Infant Death Syndrome (SIDS)

**DOI:** 10.3389/fped.2022.865051

**Published:** 2022-04-15

**Authors:** Paul Nathan Goldwater

**Affiliations:** Adelaide Medical School, Faculty of Health and Medical Sciences, The University of Adelaide, Adelaide, SA, Australia

**Keywords:** Sudden Infant Death Syndrome, SIDS, Sudden Unexplained Infant Death, SUID, pathology, epidemiology, physiology

## Abstract

This *Viewpoint* paper presents a timely and constructive critique of mainstream SIDS research. It is concerning that twenty-first century medical science has not provided an answer to the tragic enigma of SIDS. The paper helps explain why this is so and illustrates possible shortcomings in the investigation of Sudden Infant Death Syndrome/Sudden Unexplained Infant Death (SIDS/SUID) by mainstream researchers. Mainstream findings are often based on questionable and dogmatic assumptions that return to founding notions such as the Triple Risk Hypothesis and the contention that the mechanisms underlying SIDS/SUID are heterogeneous in nature. The paper illustrates how the pathological findings in SIDS have been under-investigated (or ignored) and that key epidemiological risk factors have slipped from memory. This apparent amnesia has resulted in failure to use these established SIDS facts to substantiate the significance of various neuropathological, neurochemical, or other research findings. These unsupported findings and their derivative hypotheses are therefore ill-founded and lack scientific rigor.

**Conclusion:**

The deficits of SIDS “science” revealed in this paper explain why the SIDS enigma has not yet been solved. To make progress in understanding SIDS, it is important that researchers, as scientists, uphold standards of research. Encouragement for new directions of research is offered.

## Introduction

Over the past 30 years, as a Sudden Infant Death Syndrome (SIDS)/Sudden Unexplained Infant Death (SUID) researcher, the author has followed mainstream's progress and has realized that mainstream approaches to the problem are flawed at a fundamental level. To address this, leading peer-reviewed publications were reviewed using PubMed and Google Scholar (search terms: “Sudden Infant Death Syndrome,” “SIDS”) to provide evidence as to why the SIDS enigma remains unexplained. Where possible, papers cited were selected on the basis of the senior author having published a minimum of 10 papers on the subject and that the papers concerned the prevailing hypotheses on SIDS. While this paper is a critique of mainstream research, its aim, through the arguments herein put, is to encourage SIDS researchers to reconsider their hypotheses in the hope of yielding improved research outcomes.

## Discussion

There are not many issues in medical science that lack explanation. It could be argued that current mainstream SIDS research remains at a stage not dissimilar to that of peptic ulceration in the pre-*Helicobacter* era. To achieve an understanding of a condition, respect must be paid to the essential elements of the condition: these elements include the epidemiology (e.g., risk factors), the pathology (including the laboratory findings), and the physiological findings. Based on these, a case definition is developed. The definition of SIDS has undergone several iterations (1–3). The often-used San Diego definition (3) states “the sudden and unexpected death of an infant under 1 year of age, with the onset of the lethal episode, apparently occurring during sleep, that remains unexplained after a thorough investigation including a performance of a complete autopsy, and review of the circumstances of death and the clinical history.” The definition is unhelpful in that its usefulness is limited to being exclusive. However, this definition, in extended form, is more helpful than previous ones, as it provides categories based on some pathological findings. All definitions could be misleading in relation to the reference to *sleep*. This allusion has led many researchers to explore physiological events during infant sleep, and such work has extended to investigate *arousal* mechanisms (4, 5). With very few exceptions, the results of these investigations do not refer to epidemiological risk factors or the gross pathological findings of SIDS. Therefore, these findings are unsupported in obvious ways. Death during sleep is not a prerequisite for diagnosing SIDS/SUID: cases occur in awake infants (6), although the San Diego definition (3) with its “*during sleep”* restriction would exclude these cases. Such restriction is only academic, as proof of being asleep cannot be ascertained. Additionally, it is known that SIDS occurs at all times of the day, suggesting that an infant could have been awake. Most SIDS cases occur between midnight and 0600 h (7).

A more recent development regarding the SIDS definition deemed as a new classification of SUID has surfaced, which is heavily weighted toward *asphyxia* as the underlying event (8). The study re-examined cases originally designated SIDS; a panel of reviewers ascertained that asphyxia potentially contributed to death in 40–59% of the cases based on a potentially risky sleeping environment. The authors “*suggest that SIDS not be used if a potential (but not necessarily proven) other cause of death exists*.” This should raise many questions: no mention in this article of extrathoracic petechial hemorrhages was made. Such a pathological finding would raise suspicion and strongly indicate an asphyxial mode of death. Other risk factors, such as prone sleeping and features of infection, were not examined, nor were the ages of cases reported; asphyxia appears to be uncommon in older cases than the peak age (2–4 months) of SIDS. In a similar study, Garstang et al. (9) found that only 14% of SUID could be reclassified as caused by asphyxia, which puts the findings of Randall et al. under further question.

## The Importance of Autopsy Findings

In understanding the fundamental facts about SIDS, the shortcomings of mainstream SIDS/SUID research will become obvious. The current dogma purports that the gross pathology of SIDS is unremarkable (10). This is fundamentally erroneous. As with the investigation of adult deaths the autopsy remains the mainstay for proper diagnosis (11). The same applies to the investigation of SIDS. Standardized autopsy protocols go some way in improving the investigation of sudden deaths but these, unfortunately, are not universally applied. In regard to SIDS, where autopsies have been conducted by pediatric pathologists, it is remarkable that the pathological findings (12, 13) are very consistent and can be applied to approximately 90% of cases. The gross findings include

- Intrathoracic petechial hemorrhages in and on the thymus, epicardium, and visceral pleura/lungs.
- Heavy fluid-laden lungs with early subtle acute inflammatory changes.
- Heavier than normal thymus (13, 14), brain (13–21) and liver (13, 22, 23).
- Liquid blood in the chambers of the heart (12).
- Empty bladder (12).
- Raised core temperature (24).

The autopsy extends to histopathological findings and laboratory findings. These are discussed below.

## Laboratory Data

As part of the autopsy investigation, laboratory findings can also provide clues to underlying pathogenetic mechanisms in SIDS/SUID. These include increased tissue proinflammatory cytokines IFN-alpha, TNF and IL-6 (25–30), including increased IL-6 in cerebrospinal fluid and vitreous in the eye (26, 31). Raised serum fibrin degradation products (FDPs, D-dimer) (32) provide another clue, as does lower than normal serum melatonin (33). Infection and sepsis stimulate the release of serotonin, increasing serum levels of this related hormone (34). Histopathological findings also support the infection model. These include low-grade lung inflammation (35) and/or myocardial inflammation (35) and changes typical of haematogenous shock (36) and shock-like diaphragmatic muscular degeneration (37, 38). Neuropathological features that could reflect shock include neuronal apoptosis (39) and microglial activation (40). Microbiological investigation reveals detection of bacterial toxins in SIDS tissues (41, 42), isolation of bacterial pathogens (e.g., *Staphylococcus aureus* and *Escherichia coli*) from normally sterile sites (43, 44), and despite these clues, infection and sepsis have not been widely examined in relation to most aspects of SIDS research and despite the findings of those proposing the Infection Model of SIDS (25–32, 35, 41–49).

While serotonin has been a major focus of SIDS research, the work has lacked meaningful results because there has been no or minimal supporting epidemiological or clinicopathological correlation (50). Without this, interpretation of results is impossible. In regard to serotonin levels, these are confusing; for example, some studies show raised blood levels (50), while brainstem levels of tryptophan hydroxylase and serotonin receptor binding were found to be lowered (51). This seems counterintuitive. Moreover, important correlations with SIDS risk factors could not be found in these publications.

## Physiological Clues

Also important are clinicophysiological findings (52): computer memory monitored babies have been recorded as apparent SIDS/SUID deaths. These recordings demonstrated bradycardia followed by asystole. Gasping respirations and cessation of breathing followed the cardiological events and suggest that the cause lies within the heart rather than respiratory control. Prone sleeping has been assumed to be related to asphyxia, but its likely real reason for increased SIDS risk has been overlooked *(vide infra)*.

## Triple Risk Hypothesis

In fashioning a research direction, a number of models incorporating known risk factors have been proposed and refined (53–55). These eventuated in the SIDS “triple risk” model (56, 57). It supposes that the risk of SIDS is increased when a vulnerable infant is exposed to environmental stressors. The three components of the model are (1) a critical developmental period *in homeostatic control* (from 1 to 6 months, especially 2–4 months, the “SIDS peak”); (2) exposure to stressors (overheating, infection), and (3) underlying susceptibilities (age, sex, race, etc.) (57). The model has since been modified, but its essence remains much the same (57). Guntheroth and Spiers (57) concluded after analyzing in detail the series of hypotheses… “*The advantage of any of the triple risk hypotheses in understanding SIDS has not been demonstrated.”* This warning has not been heeded, and researchers still use the triple risk hypothesis as a platform upon which they base their research. More recently, Spinelli et al. (58) errantly continue the notion of its usefulness: the authors state that it… “*assists in helping to conceptualize SIDS”* and “*continues to provide an extremely useful framework to guide current and future research.”* Of additional concern is the emphasis of the triple risk hypothesis on *homeostatic control*. This has been misleading and requires new thinking.

In seeking a homeostatic control answer, researchers tried to link apparent abnormalities in the brainstems of SIDS cases (59, 60). They found that 40–50% of SIDS babies' brainstems appeared abnormal. Some controls had similar abnormalities.

This led a quest for a common underlying pathogenetic mechanism and advanced the theory of failure in homeostatic control (breathing and/or cardiac arrhythmia) to be central to SIDS. This approach has yet to provide a definitive answer despite concerted efforts. This focus on homeostatic control has generally ignored [with a few exceptions (39)] the key clinicopathological and epidemiological findings herein set out. The physiological monitoring information clearly relates to cardiac control (52). Investigation into the heart (and potential underlying mechanisms, e.g., sepsis) is therefore appropriate. Continuation of respiratory control research without physiological evidence of an abnormality in respiratory control would deem this line of research fruitless. Evidence of chronic hypoxia in some SIDS cases (11, 61) may have led researchers to explore a respiratory-based paradigm; however, data pertaining to “chronic hypoxia” are contradictory (62) and place this paradigm on shaky ground. Consideration of and active research into other possible causes of hypoxia (sepsis being one) (62, 63) has not occurred.

## Infection and Epidemiology

Several authors have hypothesized that SIDS could be caused by a dual infection with a respiratory virus and toxigenic bacteria (22, 41–49).

The epidemiology and gross pathology of SIDS clearly demonstrate evidence for respiratory viral infection, which could possibly act as a SIDS *trigger* (48). In many studies, more than 75% of SIDS babies featured recent or active respiratory tract infections (64, 65).

In matched case-control studies, living babies showed rates of infection similar to those of SIDS and reflected the epidemiology extant at the time. Numerous studies [reviewed by Prandota et al. (66)] have attempted to demonstrate a link between respiratory infection and SIDS. These studies naturally were unable to show a difference in viral infection [and lung pathology (67)] between SIDS and controls. However, the results of the study by Bajanowski et al. (68) favored the hypothesis that respiratory viral infection could act as a trigger in SIDS. Despite the positive findings of Bajanowski et al. (68) researchers tended to discount the possible role of infection in SIDS. Regrettably, this attitude has largely continued to this day, despite all the established infection-related epidemiological features listed below:

- seasonality (the winter peak) (47, 69)
- a pronounced association with epidemic viral diseases, including influenza A (70, 71)
- Acute illness (e.g., URTI/otitis media) with symptoms present at the time of death but are not significant as a cause of death (72). Susceptibility to infection could be influenced by genetic make-up (vide infra)
- male sex (73)
- Low socioeconomic status (74), as measured by deprivation indices, overcrowding, maternal age and maternal education, etc.
- sleeping on contaminated surfaces (the parental or other shared bed (75), used mattress (76), or sofa (77)
- high birth order wherein older siblings bring viral infection home (78)
- prematurity/preterm birth (79)
- smoke exposure (80)
- lack of breastfeeding (81)
- waning maternal transplacental IgG (82)
- Overcrowding, low socioeconomic status (74, 83)
- Prone sleep position (the effect of this appears only to operate when there is a coincident infection) (84–86) *(vide infra)*.

## Prone Sleep Position and Infection

The above features uphold the infection model for SIDS. Interaction between viral respiratory tract infection, prone sleeping and secondary nasopharyngeal bacterial flora changes leading to fatal sepsis provides a simple and plausible mechanism (48, 49).

The role of infection in SIDS has been previously addressed (45, 46) and remains salient. As suggested above, the mechanism underlying SIDS/SUDI could involve an abnormal response to viral respiratory infection at a time a bacterially colonized infant becomes challenged by a bacterial toxin. An experimental model for SIDS was suggested by Nobel Laureate Peter Doherty and his colleagues: mice exposed to a virus and challenged with a staphylococcal enterotoxin died of hematogenous shock when dually exposed. Mice did not die when exposed to the single agents (87).

Except for several research groups (41, 42, 45, 48, 49, 82, 88–94), support for a role of infection has been largely unexplored by mainstream SIDS researchers. The Tasmanian SIDS Study of Ponsonby et al. (84) unaccountably failed to reawaken interest in infection. The study was able to reveal the plausibly true nature and effect of prone sleep position and showed that the risk of SIDS was increased 10-fold if a baby slept prone *when it had features of a concurrent upper respiratory tract or other viral-like illness*. In addition, the risk of prone sleeping was hardly affected if infants were apparently infection-free. The Nordic Epidemiological Study (85, 86) confirmed the Tasmanian findings and showed an even higher risk (29-fold) of *prone-plus-infection*. Mainstream SIDS researchers have failed to acknowledge or appreciate this important finding: a nearly two-decade blind spot that may have kept a solution to the SIDS problem in the dark.

Studies featuring epidemiological, sociological and pregnancy risk factors for the prone sleeping position in SIDS often showed a relationship to winter seasonality (95, 96). It is surprising that these studies overlooked the obvious connection with infection. However, other studies had no trouble making the connection (96). It is of value to quote from the latter study by Froggatt et al. (97) “Any orthodox interpretation of our results must ascribe some role to infection, mainly respiratory infection. The greatest incidence is in Belfast among the lowest socioeconomic groups and the most crowded houses, in the coldest months, with serial correlation between SUD frequency and documented major virus epidemics, and with “season”/“city” contingency. Cases in Belfast in the winter being disproportionately prevalent;” (97).

The sleeping position of babies is featured in numerous recent and current SIDS research papers. Researchers have posited (without providing supportive evidence) that prone sleep position has a causal relationship with mortality (98). Such uncorroborated statements are not scientifically acceptable and have until now remained unaddressed. SIDS occurs in supine and side sleeping infants.

## Heterogeneous Pathogenesis vs. Single Mode of Death

Another aspect of SIDS research is the dogma promoting the notion of a heterogeneous pathogenetic process. As indicated above, the Triple Risk Hypothesis (56–58) has led the approach to the SIDS problem. The hypothesis' (56–58) focuses on *homeostatic control*, and the generally accepted abiding notion that SIDS has a *heterogeneous pathogenesis* deserves further consideration. This question has been put forth in previous publications: (23, 45, 46) why do ~90% of SIDS cases have very similar gross pathological findings? The consistent finding of intrathoracic petechiae involving the thymus, pleura and heart, the unclotted/liquid heart chamber blood, the congested lungs (usually with low-grade inflammatory changes), the empty bladder, the raised core temperature, and the characteristic organ weight findings (a large thymus, brain and liver) (46) make this collective pathology an important phenomenon that could not plausibly be a coincidence. On balance of probability, *heterogeneous pathogenesis* would imply a panoply of various pathological findings and therefore several implied modes of death. Similar pathological findings in any collection of SIDS/SUID babies logically point to a *single* mortal process. Other or absent pathological findings (not conforming to the classical gross pathology of SIDS), which could include cases resulting from genetic mutations resulting in cardiac arrhythmia, etc. would be candidates for the remaining ~10% of cases that do not conform to the classical gross pathology of SIDS/SUID. The pathological picture in SIDS should be a guide for future research efforts.

## Other Unanswered Questions

These have been discussed in detail previously (99) and deserve brief revisiting. The almost universal finding of intrathoracic petechiae in SIDS stands out as a poorly investigated phenomenon. To date, there have been no transmission electron microscopy or other relevant studies to help ascertain the nature of the vasculopathy. Studies using asphyxiated animals did not provide convincing answers (99). Another almost universal finding is liquid/unclotted blood in SIDS cases. Nevertheless, only one study has investigated this (32) and revealed increased D-dimer (FDPs), strongly suggesting coagulopathy; infection is a possible primary underlying mechanism. The review by Blackwell et al. (89) provides a comprehensive overview of key findings and risk factors and how they act through inflammatory responses and their genetic control. A number of genetic polymorphisms have been shown to be related to infection and inflammatory responses, which could help explain the increased susceptibility in SIDS babies. As indicated above, ethnicity (e.g., Australian Aboriginals and Indigenous North Americans) and male sex provide evidence of increased susceptibility and, obviously, both infer a genetic link; however, these effects can be complicated by socioeconomic and other factors (e.g., smoking) (89). The finding of cardiac ion channel mutations in a small proportion of SIDS cases remains unresolved as to whether death is *with* or *due to* the genetic mutation (100).

The consistently observed organ weight changes (heavy thymus, brain and liver) in SIDS deserve fulsome investigation. Thymic enlargement suggests some perturbation of innate or adaptive immune responses wherein infection deserves special attention (22, 23).

New tools for investigation of SIDS such as the liquid biopsy, utilizing the science of proteomics to seek new molecular biomarkers may provide interesting results.

## Conclusion

SIDS research appears to have lost its way because researchers appear to have forgotten or overlooked the epidemiological risk factors and clinical pathology because these are essential pointers to the underlying cause of SIDS/SUID. It is hoped that this article is seen as a constructive critique that highlights these neglected areas and provides encouragement for fresh thinking and therefore influence future SIDS research toward a more productive course and outcome. A recently published and easily tested novel hypothesis may provide new insights into the SIDS problem for it upholds all the epidemiological features of SIDS and is consistent with the clinicopathology of the syndrome (101).

## Data Availability Statement

The original contributions presented in the study are included in the article, further inquiries can be directed to the corresponding author.

## Author Contributions

PG as the sole author, is responsible for all aspects of this paper (including conception, literature review, writing all drafts, and final version) and approved the article for publication and agrees to be accountable for all aspects of the work in ensuring that questions related to the accuracy or integrity of any part of the work are appropriately investigated and resolved.

## Conflict of Interest

The author declares that the research was conducted in the absence of any commercial or financial relationships that could be construed as a potential conflict of interest.

## Publisher's Note

All claims expressed in this article are solely those of the authors and do not necessarily represent those of their affiliated organizations, or those of the publisher, the editors and the reviewers. Any product that may be evaluated in this article, or claim that may be made by its manufacturer, is not guaranteed or endorsed by the publisher.

## Abbreviations

SIDS: sudden infant death syndrome
SUID: sudden unexplained infant death
SUDI: sudden unexplained death in infancy
IFN: interferon
IL-6: interleukin-6
FDP: fibrin degradation products.
